# A novel program of infiltrative control in astrocytomas: ADAM23 depletion promotes cell invasion by activating **γ**-secretase complex

**DOI:** 10.1093/noajnl/vdad147

**Published:** 2023-11-14

**Authors:** Elisa Helena Farias Jandrey, Gabriela Filoso Barnabé, Marcos Maldaun, Paula Fontes Asprino, Natália Cristina dos Santos, Lilian Tiemi Inoue, Andrei Rozanski, Pedro Alexandre Favoretto Galante, Suely Kazue Nagahashi Marie, Sueli Mieko Oba-Shinjo, Tiago Góss dos Santos, Roger Chammas, Carmen Lucia Penteado Lancellotti, Frank B Furnari, Anamaria Aranha Camargo, Érico Tosoni Costa

**Affiliations:** Molecular Oncology Center, Hospital Sírio-Libanês, São Paulo, Brazil; Molecular Oncology Center, Hospital Sírio-Libanês, São Paulo, Brazil; Molecular Oncology Center, Hospital Sírio-Libanês, São Paulo, Brazil; Molecular Oncology Center, Hospital Sírio-Libanês, São Paulo, Brazil; Molecular Oncology Center, Hospital Sírio-Libanês, São Paulo, Brazil; Molecular Oncology Center, Hospital Sírio-Libanês, São Paulo, Brazil; Molecular Oncology Center, Hospital Sírio-Libanês, São Paulo, Brazil; Molecular Oncology Center, Hospital Sírio-Libanês, São Paulo, Brazil; Department of Neurology, Laboratory of Molecular and Cellular Biology, LIM15, FMUSP, São Paulo, Brazil; Department of Neurology, Laboratory of Molecular and Cellular Biology, LIM15, FMUSP, São Paulo, Brazil; Centro Internacional de Pesquisa, A.C. Camargo Cancer Center, Fundação Antônio Prudente, São Paulo, Brazil; Laboratório de Oncologia Experimental, Centro de Investigação Translacional em Oncologia, Instituto do Câncer do Estado de São Paulo, São Paulo, Brazil; Departamento de Patologia, Santa Casa de São Paulo, Brazil; Ludwig Institute for Cancer Research (LICR), University of California, San Diego, California, USA; Molecular Oncology Center, Hospital Sírio-Libanês, São Paulo, Brazil; Molecular Oncology Center, Hospital Sírio-Libanês, São Paulo, Brazil

**Keywords:** ADAM family, astrocytoma, Invasion, signal transduction, γ-secretase

## Abstract

**Background:**

Infiltration is a life-threatening growth pattern in malignant astrocytomas and a significant cause of therapy resistance. It results in the tumor cell spreading deeply into the surrounding brain tissue, fostering tumor recurrence and making complete surgical resection impossible. We need to thoroughly understand the mechanisms underlying diffuse infiltration to develop effective therapies.

**Methods:**

We integrated *in vitro* and *in vivo* functional assays, RNA sequencing, clinical, and expression information from public data sets to investigate the role of ADAM23 expression coupling astrocytoma’s growth and motility.

**Results:**

ADAM23 downregulation resulted in increased infiltration, reduced tumor growth, and improved overall survival in astrocytomas. Additionally, we show that ADAM23 deficiency induces γ-secretase (GS) complex activity, contributing to the production and deposition of the Amyloid-β and release of NICD. Finally, GS ablation in ADAM23-low astrocytomas induced a significant inhibitory effect on the invasive programs.

**Conclusions:**

Our findings reveal a role for ADAM23 in regulating the balance between cell proliferation and invasiveness in astrocytoma cells, proposing GS inhibition as a therapeutic option in ADAM23 low-expressing astrocytomas.

**Key Points**

- We found a new program of infiltrative control in astrocytomas in which ADAM23 controls a Gamma-secretase (GS)-dependent pro-invasive phenotype.
- ADAM23 can be useful to identify a subclass of patients that might benefit from the use of GS inhibitors.

**Importance of the Study**

A program of infiltrative control in astrocytoma cells in which ADAM23 downregulation induces Gamma-secretase (GS)-dependent reprogramming towards a pro-infiltrative phenotype. ADAM23 is a prognostic marker in diffuse astrocytomas and can be useful to identify a subclass of patients who might benefit from the use of GSIs.

## Background

Astrocytomas are the most common and deadly tumors of the central nervous system (CNS). The World Health Organization (WHO) segregates astrocytomas into “circumscribed” (WHO Grade 1), often exhibiting a solid morphology, as opposed to the inherently “diffuse” astrocytomas (WHO Grades 2–4).^1^ The gold-standard treatments—including, radical surgery, temozolomide, and radiotherapy—for diffuse astrocytomas do not guarantee tumor eradication and results in short survival gains.^2^ One of the major causes of treatment failure is the infiltrative growth pattern of the astrocytoma cells in the brain parenchyma, hindering total tumor resection and fostering tumor recurrence.^3^ Infiltrative growth is driven by a specialized subpopulation of invasive cells called glioma stem-like cells (GSCs). Despite no effective treatment against GSCs has been established yet, various signaling pathways, including Notch, Slit/Robo, and sonic hedgehog (Shh) pathways, work cooperatively to sustain their stemness and invasiveness.^4^ Thus, elucidating the molecular mechanism underlying the infiltrative phenotype will provide crucial molecular targets for novel therapies. ADAM23 belongs to a subgroup of three catalytically inactive ADAM (A disintegrin and metalloproteinases) members (ADAM11, 22, and 23), known as “cerebral ADAMs.”^5^ It is predominantly expressed in proliferative niches of the brain and plays a crucial role in neuronal development, myelination, and differentiation.^6,7^ Several studies have shown that *ADAM23* is downregulated in many types of human cancers, including breast,^8–10^ head and neck,^11^ colorectal,^12^ lung,^13^ bone,^14^ ovarian,^15^ and brain^16^ cancers. Considering the large spectrum of human cancers associated with *ADAM23* silencing and its major expression in the brain, it is likely that *ADAM23* downregulation is associated with the activation of malignant programs in astrocytoma cells. Gamma-secretase (GS) is a transmembrane protein complex composed of two presenilin isoforms (PS1 or PS2), nicastrin (Nct), three different anterior pharynx defective-1 isoforms (Aph-1aS, Aph-1aL, Aph-1ab), and presenilin enhancer-2 (Pen-2). GS have more than a 100 putative substrates recognized, with amyloid precursor protein (APP) and Notch the best characterized.^17^

Here we showed that the gene *ADAM23* is downregulated in diffuse astrocytoma (Grade 2–4) cells compared with the normal brain tissues and that the invasive behavior of astrocytoma cells is inhibited by ADAM23. Moreover, depletion of endogenous ADAM23 in GSCs results in increased brain infiltration and slow-growing tumors, prolonging survival in patients with astrocytoma and murine models owing to reduced cerebral herniation. We identified gene expression signatures associated with Alzheimer’s disease (AD) and we found that ADAM23 depletion increases GS activity in astrocytoma cells. Interestingly, treatment with GS inhibitors (GSIs) or genetic knockdown of presenilin-1 (PS1)—the catalytic component of GS—reverts the invasive gains associated with ADAM23 depletion. Our data support that *ADAM23* downregulation in GSCs and non-GSC astrocytoma cells promotes diffuse astrocytoma invasiveness by increasing GS activity. Our results also suggest the possibility of using GSI as a new line of targeted therapeutic agents for diffuse astrocytomas with intrinsic low ADAM23 expression levels.

## Methods

### Clinical Data

Non-neoplastic brain (NNB) from epilepsy patients and WHO Grades 2–4 astrocytomas were collected from patients undergoing treatment at the Hospital das Clinicas (HC) at the Faculdade de Medicina of the University of São Paulo (FM-USP), Brazil. Validation of ADAM23 expression was performed from the Rembrandt data set (Rembrandt 2005). Clinical data on overall survival (OS) were obtained from The Cancer Genome Atlas (TCGA) and the Chinese Glioma Atlas (CGGA).

### Cells, Plasmids, and Reagents

Parental GBM6 and GBM39, GSC23 and GSC11, and HK296 and HK301 cells were cultured in DMEM/F12 medium supplemented with 2% B27 (GIBCO/Life Technologies) and 20 ng/ mL human EGF and bFGF, and 2 mg/mL Heparin. U87MG, U178MG, and U343MG cell lines were maintained in DMEM + 10% FBS. All cells were incubated at 37°C with 5% CO_2_ in a humidified incubator and were certified to be Mycoplasma-free. PLKO.1-based ADAM23, (IPTG)-inducible (pLKO_IPTG_3xLacO) and PS1 shRNA constructs were purchased from Sigma (Mission shRNA). Cells were treated with 10 µM of GSI RO4929097 (Stem Cell Technologies) for 24 h. For determination of GS activity, cells were subjected to Aβ ELISA immunoquantification (aa1-42, DAB142 R&D Systems).

### Orthotopic Transplantation

Viable 10^4^ GSC23 or U87 cells in 3 μl of medium without growth factors were stereotactically injected into the right striatum of 8-week-old female nude mice, as previously described.^18^ Tumor growth was assessed every two weeks by iVIS Spectrum In Vivo Imaging System (PerkinElmer). Mice were sacrificed at the time of development of neurological symptoms or body weight loss > 20% by isoflurane inhalation (4–5 vol.%) followed by inhalation of CO_2_.

### Immunohistochemistry and Immunofluorescence

Immunohistochemistry (IHC) was performed using the EXPOSE kit following the manufacturer’s instructions (Abcam, Ab80436). Reactions were performed with the primary anti-Sox2 antibody (Cell Signaling, clone D6D9, Cat # 3579), anti-Aβ, and anti-ADAM23 (Sigma-Aldrich Co., HPA012130). Immunofluorescence (IF) was performed in monolayers of U87 cells or in GSC neurospheres fixed in 4% formaldehyde for 10 min, washed and blocked with 0.1% Triton-X, 2% BSA, and 1% FBS for 15 min at 4°C. The cells were incubated with an anti-Cleaved Notch1 monoclonal antibody (D3B8, #4147, Cell Signaling) for 1 h at room temperature. Detection was performed with Alexa Fluor-labeled secondary antibodies (Molecular Probes). Images were taken under a DS-Fi2 (Nikon) digital camera microscope coupled.

### TCIA-TCGA Data Set

Preoperative astrocytoma patients from TCGA who had gene expression profiles and corresponding MR imaging available in the NCI’s TCIA (http://cancerimagingarchive.net/). We used Fluid-Attenuated Inversion Recovery (FLAIR) sequences that reflect a mixture of edema and tumor infiltration and are routinely used to evaluate its extent. FLAIR sequence volumes and 3D reconstructions were acquired in 3D slicer software 4.10 version.^19^

### Time-Lapse Microscopy of Cell Migration

Individual cell migration was monitored on EVOS FL-AUTO 2 equipment (Thermo Fisher Scientific) equipped with a temperature and gas supply control. The cell motility was measured by using the “Manual Tracking” and “Chemotaxis and Migration Tool” (Ibidi) plugins from ImageJ software (NIH, Bethesda, MD, USA).

*Invasion assays.—*The 2 × 10^5^ cells were plated in the upper chamber of the matrigel-coated transwell and allowed to invade for 22 h at 37°C. 600 μl of the complete medium was used as a chemo-attractant into the bottom of the lower chamber. Cell invasion analysis in 3D purified brain matrix or 3D matrigel was performed as described previously.^20^

### Fluorescence Molecular Tomography

Intracranial fluorescence images were obtained by the *in vivo* imaging systems (IVIS SpectrumCT) and were used to indirectly track the GSC invasion as previously described.^21^

### RNA-seq

Total RNA was enriched for polyA+-containing RNA using oligo-dT dynabeads and then used as a template for cDNA synthesis. Library products were submitted to sequencing in the Illumina platform using the protocol “Hi-Seq Rapid Run Duo SR 100 Cycle.” Read sequences were aligned and mapped against the human genome using RNA Express Application (v1.0.0) from BaseSpace.

### scRNAseq

The human brain data set from Darmanis et al.^22^ was downloaded from Gene Expression Omnibus (GEO) with accession number GSE84465.

### Fractal Analysis

Images were processed using ImageJ software (NIH, Bethesda, MD, USA). Briefly, gray-scale images were evaluated for the fractal dimension using the box-counting method provided by the Multifrac plugin of ImageJ software.^23^

### Statistical Analyses

The results obtained were tested for normality using the Shapiro–Wilk test. Parametric data (*P* > .05) were expressed as mean ± standard deviation (SD) and analyzed using the unpaired t-test or one-way ANOVA followed by Bonferroni’s post-test.

## Results

### *ADAM23* Gene Expression Is Downregulated in Diffuse Astrocytomas and Promotes Brain Infiltration

**Figure 1.**
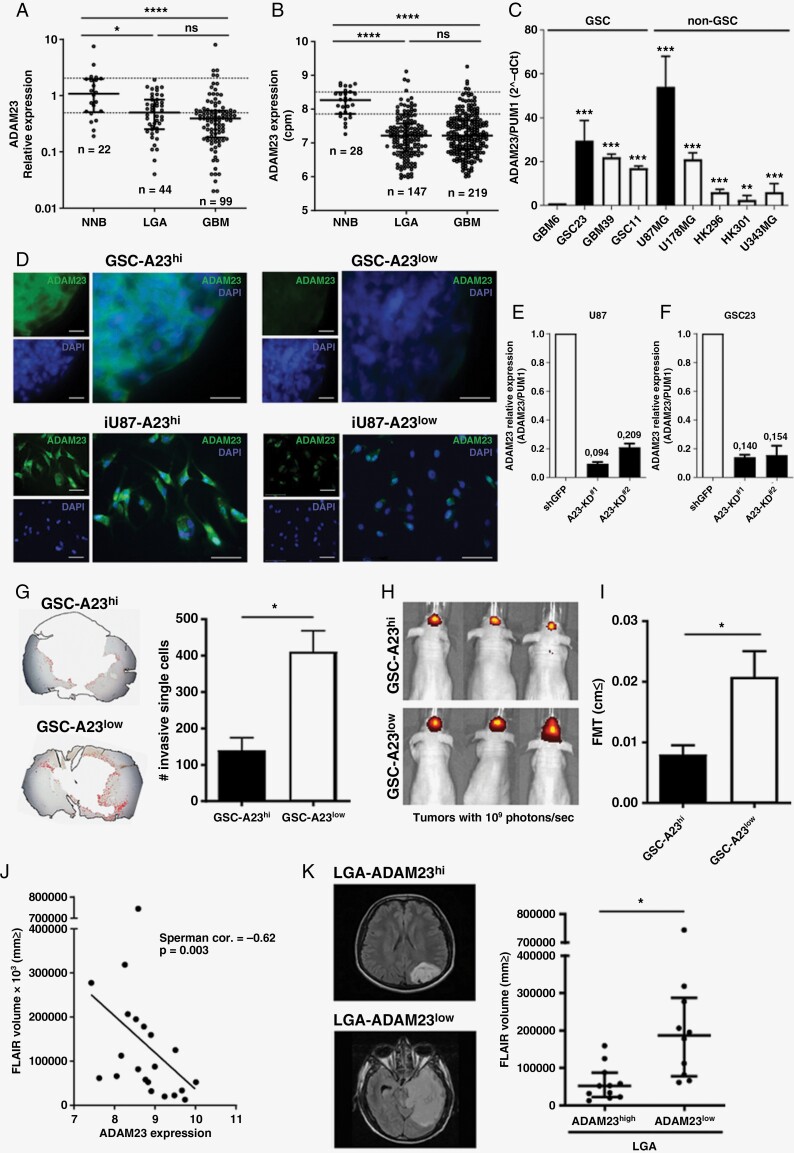
ADAM23 depletion increases astrocytoma infiltration. (**A**) mRNA expression analysis of ADAM23 from **(A)** HC cohort (*n* = 143) and (**B)** from REMBRANDT (*n* = 394). (**C**) RT-qPCR analysis of ADAM23 mRNA expression in several GSC and GBM cell lines. (**D**) Representative images of Adam23 expression in GSC23 and U87 cells before (A23^hi^) and after ADAM23-depletion (A23^low^). (**E**) RT-qPCR analysis of ADAM23 mRNA expression in GSC23 and **(F)** U87 cells after ADAM23 depletion. Data is represent as mean ± SD (*n* = 3). **(G)** Representative IHC staining and quantification of GSC invading the normal brain parenchyma of mice. The number of invasive single TurboFP635/Sox2-positive human cells were artificially colored. To quantify the number of invasive cells we first exclude the tumor core from image (white area at the center of the picture) and the number of human cells (red colored) was manually counted using the “Cell Counter” plugin of ImageJ. Data is represent as mean ± SD (*n* = 3 mice for each condition). (**H**) Representative pictures of GSC tumors and **(I)** brain infiltrative evaluation by fluorescence molecular tomography (FMT; *n* = 3 A23^hi^ and *n* = 6 A23^low^). (**J**) Correlative analysis of ADAM23 mRNA expression (TCGA) and MRI FLAIR sequences (TCIA) in 21 LGA (ADAM23^low^ = 10 and ADAM23^hi^ = 11) (spearman correlation = -0.62, *P* = .003). **(K)** Representative MRI FLAIR sequences showing a LGA ADAM23^hi^ (left) and a LGA ADAM23^low^ (right) tumor. *P < .05 and ****P < .0001.

*ADAM23* transcripts are expressed in several normal tissues with particularly high levels in multiple regions of the CNS^24^ (Supplementary Figure 1). To evaluate *ADAM23* expression in CNS tumors, we measured ADAM23 mRNA levels using qRT-PCR in the non-neoplastic brain (NNB) and 143 WHO Grades 2–4 astrocytomas from the HC cohort. *ADAM23* expression was significantly lower in diffuse astrocytomas compared with NNB (Figure 1A, *P* < .001). Downregulation of *ADAM23* expression in CNS tumors was validated in the public Rembrandt database. Independent of the tumor grade, all astrocytomas displayed a marked decrease in *ADAM23* expression compared with NNB (Figure 1B, *P* < .0001).

Next, to assess the role of *ADAM23* expression in astrocytomas, we examined ADAM23 levels in a panel of GSC and non-GSC GBM cell lines (Figure 1C, *P* < .001). GSC23 and U87 cells amongst the glioma cells had the highest expression levels of *ADAM23*, as confirmed using IF assays (Figure 1D). GSC23 and U87 cells were transduced with two independent shRNAs against *ADAM23* (hereafter named GSC- or U87-A23^low^ cells) or a control hairpin against GFP (hereafter named GSC- or U87-A23^hi^ cells) (Figure 1E and F, *P* < .001) and orthotopically grafted into nude mice. Qualitative blind microscopic examination by an expert neuropathologist revealed that GSC23 tumors were remarkably infiltrative, with GSC cells detected in both hemispheres with a partial recapitulation of key histological features of human GBM (Supplementary Figure S2). In contrast, orthotopic xenografts of U87 cells that typically exhibit a noninfiltrative growth pattern in the brain parenchyma did not exhibit any infiltrative gains or significant changes in their histopathology, even after ADAM23 depletion (Supplementary Figure S3A and B).

GSC23 infiltration showed a 3-fold increase in the number of disseminated cells in GSC-A23^low^ tumors compared with GSC-A23^hi^ tumors (Figure 1G). Additionally, to indirectly track the dispersion of GSC-turboFP635+ cells into the healthy brains, specific ROIs (regions of interest) were drawn from fluorescence molecular tomography (FMT) images to encompass the total area of the brain that is covered by GSC-turboFP635+ cells. ROIs measurements showed that GSC-A23^low^ tumors significantly spread over a larger area of the mouse brain compared with the GSC-A23^hi^ tumors (Figure 1H and I, *P* < .05).

The role of *ADAM23* in the infiltrative behavior of astrocytomas was evaluated using 21 preoperative astrocytoma data sets included in the TCIA-TCGA repositories. Subgroup cases (ADAM23^hi^ and ADAM23^low^) were compared to determine the fluid-attenuated inversion recovery (FLAIR) signal volume images that matched the level of peritumoral invasion/edema. Interestingly, these neuroimaging findings and *ADAM23* expression were inversely correlated (Spearman correlation = −0.62, *P* = .003, Figure 1J). We observed a 3.8-fold higher FLAIR volume in ADAM23^low^ low-grade astrocytomas (LGA, Figure 1K, *P* < .05). Accordingly, we suggest that lower levels of *ADAM23* may facilitate the emergence of a brain infiltrative tumor phenotype in preclinical models and human patients.

### ADAM23 Regulates Astrocytoma Cell Migration and Invasion *In Vitro*

**Figure 2.**
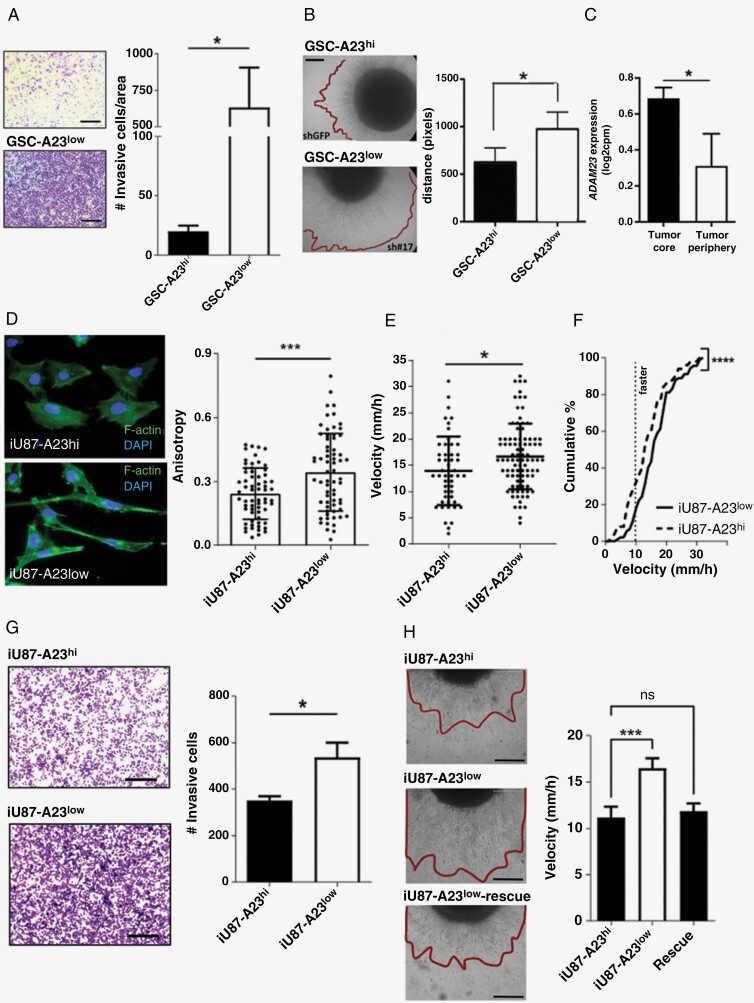
Low ADAM23 expression induces migration and invasion in malignant astrocytomas cells. (**A**) Invasive GSC-A23^hi^ and -A23^low^ cells in a transwell invasion assay. Data is presented as mean ± SD (*n* = 3). **(B)** Representative images of GSC neurospheres embedded in 3D Matrigel after 3 days. Data is represent as mean ± SD (*n* = 3-10 neurospheres/condition). **(C)** Intratumoral heterogeneity of ADAM23 expression between the invasive front and tumor core in GBM clinical samples using scRNA-seq data set.^22^ Data is representing as mean ± SD. *P*-value from two-tailed student *t-*test **(D)** Representative images of F-actin fibers and DAPI in iU87 cells plated on B-ECM and treated with IPTG for 4 days. Anisotropy evaluation using ImageJ/FIJI Fibril tool plugin in 64 iU87-A23^hi^ cells and 66 -A23^low^ cells. **(E)** Median velocity (μm/h) of iU87-A23^hi^ and -A23^low^ cells (IPTG 1mM for 4 days) plated on B-ECM. **(F)** Cumulative percentage of iU87-A23^hi^ and -A23^low^ cells in each velocity. **(G)** Representative images and count of invasive iU87-A23^hi^ and -A23^low^ cells cultured in the presence of IPTG for 4 days in a transwell invasion assay. Data is represent as mean ± SD (*n* = 3). **(H)** Representative images of multicellular spheroid invasion in B-ECM 3D hydrogel. The assay was performed with iU87-A23^hi^ (no IPTG), -A23^low^ (plus 1mM IPTG for 4 days) and -A23^low^-Rescue (after 20 days of IPTG washing) cells. Data is represent as mean ± SD (*n* = 5-8 spheroids/condition). * p < 0.05, *** p < 0.001. ns = non significant.

To better characterize the functional consequences of ADAM23 depletion, we used a Matrigel-based transwell invasion assay. A significant 16-fold increase in the invasion of 3D matrices was observed for GSC-A23^low^ cells compared to GSC-A23^hi^ cells (Figure 2A, *P* < .05). Complementarily, 3 days after uniform GSC-A23^hi^ and -A23^low^ neurospheres are embedded in a 3D Matrigel, we observed that ADAM23 depleted cells invaded distances 75% greater than ADAM23^hi^ cells (Figure 2B, *P* < .05). Considering that ADAM23^low^ astrocytoma cells can travel over longer distances than ADAM23^hi^ cells, we investigate the spatial heterogeneity of ADAM23 in clinical samples using single-cell RNA-seq data set.^22^ Our analysis revealed that ADAM23 expression is associated with an intratumor compartment of origin: being 2.2-fold downregulated at the invasive front of GBM compared to core GBM cells (Figure 2C, *P* < .05).

Next, ADAM23-shRNAs were placed under the control of an IPTG-inducible promoter in U87 cells (iU87 cells; Supplementary Figure 4H, *P* < .001). Three hours after seeding on brain extracellular matrix (B-ECM), the F-actin stress fibers revealed that IPTG-induced iU87 cells exhibit an intense cytoskeleton remodeling with a significant 40% increase in the F-actin stress fiber anisotropy (ie, alignment of stress fibers), exhibiting a mesenchymal-like morphology with an evident front/back polarity when compared to the non-induced cells (Figure 2D, *P* < .001). Twenty-four hours after seeding on B-ECM, IPTG-induced iU87 cells exhibited a 25% increase in migration speed (Figure 2E, *P* < .05), with up to 82% of cells switching to a faster migratory mode (>10 µm/h; Figure 2F, *P* < .0001), 13% increase in total distance, and 45% in directional movement (Supplementary Figure 4A and B) when compared with control cells (ie, U87 cells transduced with IPTG-inducible control-shRNAs). Complementarily, 4 days after exposure to IPTG we observed a 52% increase in the number of cells that invade the 3D Matrigel (Figure 2G, *P* < .05). When iU87 cells cultured as multicellular spheroids were embedded into B-ECM, we observed a 48% increase in the matrix invasion speed after IPTG treatment, that is completely restored to pre-IPTG (basal) levels at Day 15 of IPTG withdrawal. (Figure 2H, *P* < .001).

Interestingly, acute depletion of ADAM23 (8–10 days) induced G0/G1 cell cycle arrest (Supplementary Figure S4C, *P* < .001) and significantly reduced the proliferation and clonogenicity of iU87 cells (Supplementary Figure S4D and E). Remarkably, the downregulation is reversible after withdrawal of the IPTG, as observed by the reappearance of the ADAM23 levels (Supplementary Figure S4F) and a restoration of the original proliferative phenotype (Supplementary Figure S4D). This suggests that the ADAM23-dependent invasive/proliferative switch is in a reversible state.

### ADAM23 Depletion Promotes Extension in Overall Survival Associated With Decreased Brain Herniation

**Figure 3.**
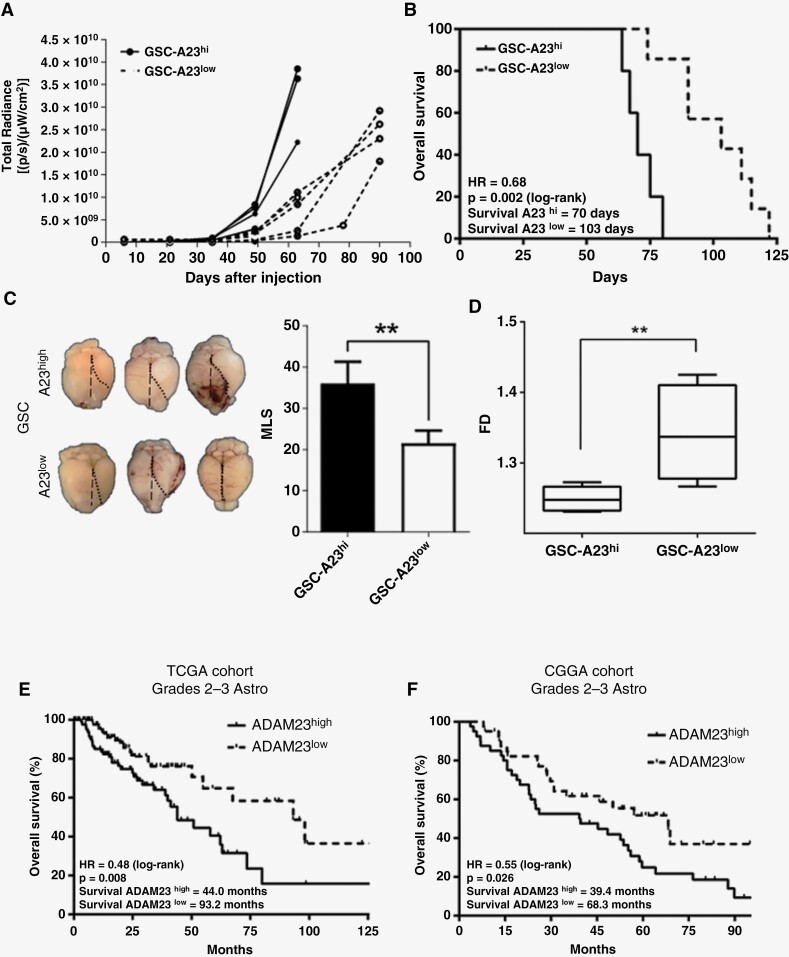
**ADAM23 depletion increases OS. (A)** Kinetics of individual GSC tumor growth in Balb/c nude mice inoculated with 1,500 ADAM23^hi^ (A23^hi^) or ADAM23^low^ (A23^low^) GSCs. **(B)** Kaplan-Meier overall survival curves of nude mice bearing GSC-A23^hi^ and GSC-A23^low^ tumors. Log-rank test, *P* = .002. **(C)** Representative pictures of the excised mouse brain samples showing the midline shift (MLS) caused by the growth of intracranial GSC tumors. Data is representing as mean ± SD. **(D)** Fractal dimension values (FD) using the box-counting method on histopathological slices of mice brain to quantify the morphological complexity of individual GSC tumors. **(E-F)** Kaplan-Meier overall survival curves of LGA (Grade 2–3) patients’ from TCGA and CGGA cohorts dichotomized in ADAM23^low^ and ADAM23^high^ groups. * *P* < .05, ** *P* < .01.

Although no significant differences were observed in the tumorigenic potential, latency, or final tumor sizes, we observed a slower tumor growth rate and an extension of OS in mice bearing GSC-A23^low^ tumors (Figure 3A-B, *P* = .002 and Supplementary Figure S5). At euthanasia, comparing same-size tumors in both groups (ie, at d65 for GSC-A23^hi^ and d103 for -A23^low^ tumors), blind microscopic examination by an expert neuropathologist did not detect any significant histological differences (necrosis, hypercellularity, edema, etc.; data not shown), but macroscopic observations reveal that GSC-ADAM23^low^ tumors exerted a significant 40% reduction in the brain midline shift (MLS) compared to same-size GSC-ADAM23^hi^ tumors (Figure 3C). These data suggest that mass effect due to growing tumor masses is impacted by ADAM23 expression levels. To better estimate this latter assumption, we used the fractal dimension (FD) using the box-counting method on histopathological slices of mice brains to quantify the morphological complexity of individual tumors. The results revealed that FD is up to 7% higher in GSC-A23^low^ tumors in comparison to GSC-A23^hi^ tumors, supporting our hypothesis that ADAM23^low^ tumors tend to be better space-filling tumors, leading to a reduction of the displacive forces in the adjacent brain structures (ie, low MLS; Figure 3D, *P* = .009).

Next, to determine whether ADAM23 levels might be a biomarker of improved survival in human astrocytomas, we used the clinical and expression information from the TCGA and CGGA cohorts. Patients were grouped according to their median *ADAM23* expression into high and low groups. The OS was not different between patients with ADAM23^hi^ and ADAM23^low^ GBM (Figure 3C, TCGA: HR = 1.26 [0.55–1.12], *P* = .188, and CGGA: HR = 0.95 [0.71–1.26], *P* = .71). Interestingly, subjects with ADAM23^hi^ low-grade astrocytomas (LGA, Grades 2–3) had a worse prognosis than those with ADAM23^low^ ones (TCGA: HR = 0.49 [0.28–0.82], *P* = .008; CGGA: HR = 0.55 [0.32–0.92], *P* = .026) (Figure 3E and F) independently of IDH status (Supplementary Figure S6) and successfully recapitulating the differences in OS observed in mouse model (Figure 3B).

### ADAM23 Depletion Reprograms Astrocytoma Cells to a Pro-Infiltrative and Alzheimer’s Disease-Like Gene Expression Signature

**Figure 4.**
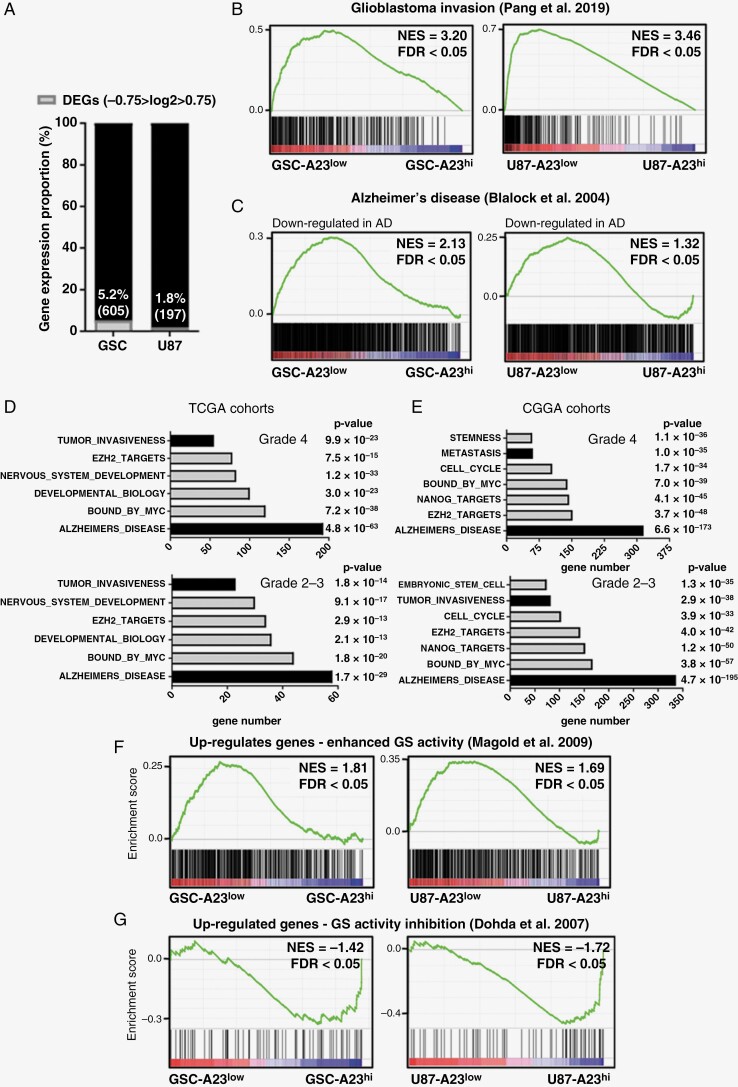
Low ADAM23 expression in malignant astrocytomas correlates with invasive gene and AD gene signatures. (**A**) RNA-seq data analysis showed the proportion of DEGs in GSC and U87 cells before (A23^hi^) and after (A23^low^) ADAM23 depletion (−0.75 < log2 > 0.75, *q*-value < 0.05). GSEA analysis comparing A23^hi^ vs. A23^low^ groups using (**B**) an invasive signature^24^ or (**C**) the AD signature.^25^ (**D, E**) Upregulated processes correlated with ADAM23 depletion levels in GBM (**D**) and LGA (**E**) using TCGA and CGGA cohorts. (**F**) GSEA analysis of RNA-seq data from GSC and U87 cells comparing A23^hi^ versus A23^low^ groups using γ-secretase activity signature^26^**(G)** GSEA analysis of RNA-seq data from GSC and U87 cells comparing A23^hi^ versus A23^low^ groups using a signature associated with the GSI DAPT.^27^

Next, gene expression profile analysis (RNA-seq) was performed to identify differentially expressed genes (DEGs) associated with *ADAM23* status in GSC and U87 cells. We found that 5.2% (605/11 591) and 1.8% (197/10 967) of the total genes were DEGs in GSCs and U87 cells, respectively, after ADAM23 depletion (Figure 4A, −0.75 ≥ log2FC ≥ 0.75, FDR < 0.05). Only seven DEGs, including ADAM23, were differentially expressed in both cell lines (*NMB*, *GLTSCR2*, *MBNL3*, *CLIP2*, *TMEM134*, and *SLCO4A1*; Supplementary Figure S7). Despite the low correspondence of DEGs in GSC and U87 cell lines, GSEA analysis revealed that the GSC and U87 *ADAM23*^low^ cell transcriptional states significantly matched the biological processes associated with GBM invasion^24^ (Figure 4B, GSC NES = 3.20, FDR *q*-value < 0.05; U87 NES = 3.46, FDR *q*-value < 0.05) and signaling pathways associated with AD^25^ (Figure 4C, GSC NES = 2.13, FDR *q*-value < 0.05; U87 NES = 1.32, FDR *q*-value < 0.05).

To extend these observations to patient data, gene co-expression analysis was used to identify regulatory networks associated with ADAM23 levels in LGA and GBM from two additional cohorts (TCGA and CGGA). Accordingly, GO annotations of gene sets inversely correlated with *ADAM23* mainly included terms related to tumor invasiveness and AD (Figure 4D and E). We also identified significant enrichment for genes associated with enhanced GS complex activity^26^ in ADAM23^low^ astrocytoma cells (Figure 4F, GSC NES = 1.81 and U87 NES = 1.69, FDR *q*-value < 0.05). Consistently, genes upregulated in response to pharmacological GS inhibition^27^ were significantly enriched in ADAM23^hi^ astrocytoma cells (Figure 4G, GSC NES = −1.42 and U87 NES = −1.72, FDR *q*-value < 0.05).

### ADAM23-Depletion Increased γ-Secretase Complex Activity

**Figure 5.**
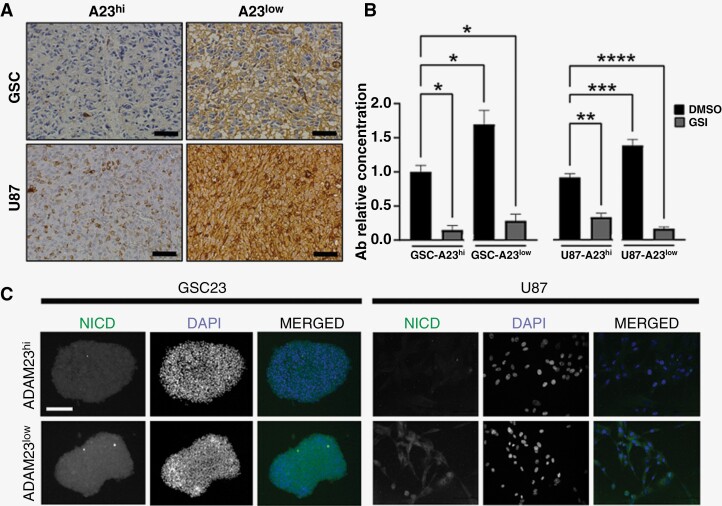
ADAM23 depletion induces γ-secretase enzymatic activity in malignant astrocytomas. (**A**) Representative IHC staining of Aβ expression in intracranial tumors of GSC and U87 cells. Bar represents 25 µm. (**B**) Quantification of Aβ in the supernatant of iU87 (left) and GSC (right) cells comparing A23^hi^ versus A23^low^ groups treated with GSI (10 µM) for 24 h. Data is represented as mean ± SD (*n* = 3). One-way ANOVA test shows there is a significant difference, with a *P < .05 **(C)** Representative images of NICD1 in GSC and U87 cells. *P < .05, **P < .01, *** *P* < .001, **** *P* < .0001. Bar represents 50 µm.

Aiming to uncover the effects of ADAM23 depletion on GS activity, we overlapped well-known 90 GS substrates^28^ with mRNAs constitutively expressed by GSC23 and U87 cells. Seventeen of these genes (19%), including: APP, NOTCH1, SDC2, LRP1, ROBO1, EFNB2, DAG1, CDH2, LRP6, APLP2, PAM, KCNE4, VLDLR, CLSTN1, SORT1, APLP1, and DSG2, are expressed in U87 and/or GSC and ten of them (59%, 10/17) have been implicated in morphogenesis involved in neuron differentiation based on GO analysis. Next, as monitors of GS-dependent proteolysis, we focus our analysis on the generation of soluble Aβ and nuclear NICD1 peptides from APP and Notch1, respectively. We observed that ADAM23^low^ tumors increase Aβ deposits by up to 55% in GSCs and 140% in U87 tumors compared with ADAM23^hi^ tumors (Figure 5A, *P* < .05), and a 50−60% increase in Aβ levels in ADAM23^low^ cells compared with ADAM23^hi^ cells supernatants *in vitro* (Figure 5B, *P* < .0001). In both lineages, this increase was significantly reduced upon pre-treatment with a potent GSI (RO4929097) (Figure 5B, *P* < .0001). In agreement, following ADAM23 depletion the cytoplasmic levels of NICD1 increased 20% in U87 and 190% in GSC, compared with their corresponding controls (Figure 5C). Despite increased NICD, no significant differences were found for Notch canonical target genes (HES1 and HEY1; data not shown) or enrichment for NICD-target 52-gene signature^29^ (Supplementary Figure S8). These data demonstrated that ADAM23-dependent GS activity increases Aβ deposits and releases NICD, but transcriptional targets of NICD are not turned on when ADAM23 is downregulated.

### γ-Secretase Inhibition Abrogates ADAM23-Induced Invasion

**Figure 6.**
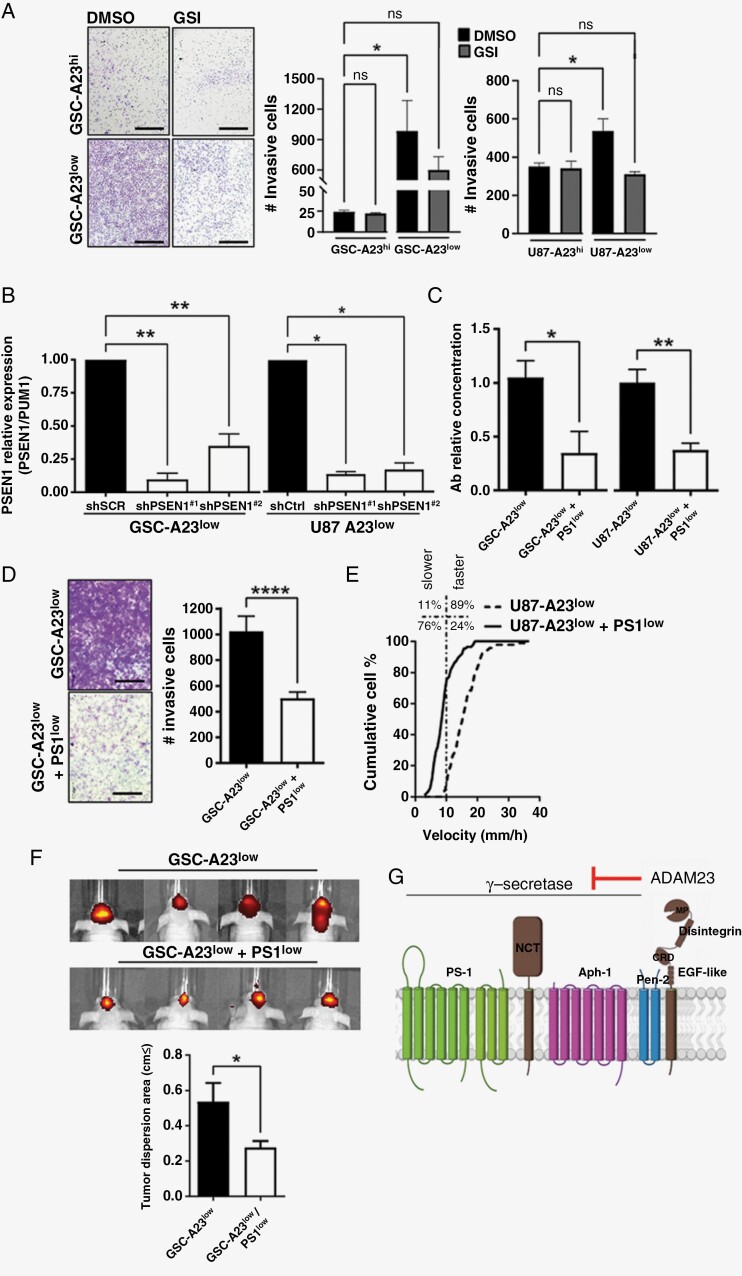
Low ADAM23 expression confers sensitivity to GSI. (**A**) Representative images and count of invasive GSC and U87 cells (A23^hi^ or A23^low^) cells treated with or without GSI 10 µM for 24h in a transwell invasion assays. One-way ANOVA test shows there is a significant difference of invasive behavior after GSI treatments specifically on ADAM23^low^ cells, with a *P < .05 (**B**) Psen-1 relative expression by RT-qPCR in GSCs and U87 cells harboring ADAM23 (A23^low^) and PS1 constitutive co-silencing (A23^low^ + PS1^low^). PS1 expression was normalized by PUM1 gene in both cell lines. (**C**) Quantification of Aβ in the supernatant of GSC (left) and U87 (right) cells silenced for ADAM23 vs. co-silenced for ADAM23 and PS1 genes. Data is represented as mean ± SD (*n* = 3). (**D**) Representative images and count of invasive GSC-A23^low^ and GSC-A23^low^/PS1^low^ in a transwell invasion assay. Data is represented as mean ± SD (*n* = 3). **(E)** Cumulative percentage of U87-A23^low^ and U87-A23^low^/PS1^low^ cells in each velocity. ****P < .0001 (**F**) Intracranial GSC-A23^low^ and GSC-A23^low^/PS1l^ow^ tumors and brain infiltrative evaluation by fluorescence molecular tomography (FMT; A23^low^*n* = 5; A23^low^ + PS1^low^*n* = 8). *P < .05. (G) Schematic diagram of the membrane topology of the ADAM23 and GS.^5^ MP (Metalloprotease domain), Disintegrin, CRD (Cysteine-rich domain) and EGF-like are extracelular domains of ADAM23. GS complex is a multiprotein complex composed of: PS-1 (presenilin-1), NCT (nicastrin), Aph-1 (anterior pharynx defective 1) and Pen-2 (presenilin enhancer).

Transwell invasive assays with ADAM23^low^ and ADAM23^hi^ cells in the presence of GSI were performed to determine whether astrocytoma cell invasion is dependent on GS activity. Interestingly, following GSI treatments we observed inhibition of over 40-50% of invasive behavior, specifically on ADAM23^low^ cells, with no effect on the invasiveness of their respective ADAM23^hi^ cells, implying that only ADAM23^KD^-dependent increment of invasion requires GS activity (Figure 6A, *P* < .05). In other words, the differential sensitivity to GSI does not stem from any particular molecular characteristic of GSCs or U87 cells (in both, invasive potential of ADAM23^hi^ cells are not sensitive to GSI), but rather it derives from ADAM23 knockdown, supporting the thesis that GS is activated by the downregulation of ADAM23 in astrocytoma cells.

The same pattern was observed in U87 and GSC ADAM23^low^ cells transduced with 2 independent shRNAs against PS1, the catalytic subunit of the GS complex (A23^low^/PS1^low^; Figure 6B, *P* < .01). Moreover, ADAM23^low^/PS1^low^ cells secreted 70% less Aβ in the supernatant (Figure 6C, *P* < .01) and exhibited significant impairment of 50% of the enhanced invasive phenotype relative to their respective scramble shRNA controls (Figure 6D, *P* < .0001). Next, we monitored the migratory behavior of U87 cells depleted of PS1 by time-lapse microscopy and observed that only 24% of U87 cells are fast-migratory cells compared with 89% of the original U87-ADAM23^low^ population (>10 µm/h, Figure 6E, *P* < .0001). Extending these observations *in vivo*, the infiltrative gain described for GSC ADAM23^low^ tumors (Figure 1G) was no longer observed after PS1 depletion (Figure 6F, *P* < .05). In addition, no differences in tumor growth rates or mouse OS were observed between the ADAM23^low^/PS1^low^ and ADAM23^low^/PS1^hi^ control groups (Figure S9A-C). Taken together, these data suggest a mechanism where GS is activated by the downregulation of *ADAM23* in astrocytoma cells. Moreover, ADAM23 depletion increased the sensitivity to GSI.

## Discussion

Despite the diversity of pathways in which GS participates, GS inhibitors (GSIs) have been repurposed as anti-cancer agents mainly due to their role in inhibiting Notch1-4 receptors cleavage, down-regulating the Notch signaling pathway.^30–32^ Preclinical studies have shown that GSIs drive GSCs into differentiated states, sensitizing them to TMZ, becoming increasingly evident in cells with higher Notch signaling activity.^32,33^ However, the results from clinical studies showed that GSIs had limited clinical benefit in most solid tumors, with few exceptions in astrocytomas.^34^ For example, a combination of GSI plus TMZ and radiotherapy demonstrated a trend toward decreased intratumoral Ki67 staining, a significant decrease in the number of NICD1-positive cells, and vascular normalization following GSI administration in patients with diffuse astrocytomas.^35^

Here, we showed that ADAM23 gene depletion in GSCs induces the characteristic transcriptional signatures of AD associated with enhanced GS activity, significantly increasing 16-fold the invasion through brain matrices *in vitro* and *in vivo*. To evaluate the ADAM23-dependent activity of GS, we choose two of the most notable endogenous GS substrates: APP and Notch. ADAM23 depletion leads to 140% increase in cerebral Aβ-amyloidosis and up to 190% increase in cytoplasmic NICD compared with ADAM23^hi^ astrocytoma cells. Despite elevated NICD, transcriptional targets of NICD are not turned on when ADAM23 is downregulated, indicating that NICD activity is subject to additional regulation.^36,37^ Although disappointing from the Notch pathway point of view, these data support the possibility that GSIs can be useful by targeting the ADAM23-dependent motility of GSCs, independently of their inhibitory role through the Notch pathway. Additionally, other well-known GS substrates, such as ROBO1, DSG-2, and Ephrin-B2, were also found expressed at lower levels in GSC and U87 cells (data not shown), and they also have the potential to be functionally associated with ADAM23-dependent phenotypes in preclinical models.^38–40^

Although preliminary, three essential aspects of ADAM23 expression in brain tissues can provide insights into the functional relevance of ADAM23 in clinical samples: (1) ADAM23 is downregulated in diffusely infiltrating astrocytomas relative to normal brain, (2) ADAM23 is downregulated at the invasive front of GBM relative to the tumor core, and (3) ADAM23 downregulation is correlated with higher FLAIR volumes, a manifestation of a combination of increased tumor infiltration and edema. So, reviewing the literature for ADAM23 promoter methylation patterns in other malignancies,^10,11^ we can suggest that ADAM23 may be also gradually and progressively downregulated during astrocytoma progression facilitating the infiltrative phenotype.

Equally important, ADAM23 knockdown decreases proliferation and delays tumor growth, while increasing survival in mouse hosts and LGA. Previously, we have reported that the intratumoral clonal heterogeneity of ADAM23 expression increases the metastatic potential of the whole tumor, due to mutual interactions between ADAM23-positive and ADAM23-negative tumor cells.^8^ In the current study, considering the existence of differential ADAM23 levels in different niches, it is plausible to speculate that the scenario is similar in astrocytomas: in the tumor core, you need an ADAM23^hi^ state to sustain chronic proliferation, while in the infiltrative front, you need an ADAM23^low^ state to support the invasion and accomplish infiltrative growth. Thus, it is reasonable to speculate that these two factors together—higher brain infiltration and slower growth—can actively modify the final tumor morphology. We showed that ADAM23 depletion leads to brain tumors with more complex morphology (7% higher FD) and with low intracranial pressure (40% lower MLS), leading us to infer that ADAM23^low^ astrocytomas are better space-filling tumors. The reduction of the compression forces exerted by these tumors—the so-called mass effect—is widely recognized as an important factor in the death of brain tumor patients,^41^ resulting in significant positive effects on OS.

Finally, our novel finding that ADAM23 regulates GS activity in astrocytomas can perhaps be attributed to direct regulatory interactions between them, similarly as observed for GS and other members of the ADAM family inside microdomains of the plasma membrane.^42^ It has been reported that most mature ADAM23 is enriched in lipid rafts^43^ and a variety of studies showed that cleavage of APP to release Aβ by GS is lipid raft-dependent as well.^44,45^ So, although the exact mechanism by which ADAM23 depletion activates GS is still unclear, we speculate that these microdomains can serve as “scaffolds” to concentrate mature ADAM23 and GS (Figure 6G).

Finally, the understanding of therapeutic vulnerabilities in astrocytomas will foster the development of more effective treatments. Our discoveries suggest that ADAM23 downregulation in astrocytoma cells activates GS promoting the proneural-mesenchymal transition (PMT) and providing an experimental basis for the use of GSI for astrocytoma patients with ADAM23^low^ tumors.

## Supplementary Material

vdad147_suppl_Supplementary_Figure_S1

vdad147_suppl_Supplementary_Figure_S2

vdad147_suppl_Supplementary_Figure_S3

vdad147_suppl_Supplementary_Figure_S4

vdad147_suppl_Supplementary_Figure_S5

vdad147_suppl_Supplementary_Figure_S6

vdad147_suppl_Supplementary_Figure_S7

vdad147_suppl_Supplementary_Figure_S8

vdad147_suppl_Supplementary_Figure_S9

vdad147_suppl_Supplementary_Figures_Legends

## Data Availability

The accession code for the RNA-seq data deposited to ENA is PRJEB52539. Additional data are available from the corresponding author upon reasonable request.
