# Supplementary figures and images for: A novel program of infiltrative control in astrocytomas: ADAM23 depletion promotes cell invasion by activating γ-secretase complex
Source: Neurooncol Adv. 2023 Nov 14;5(1):vdad147. doi: 10.1093/noajnl/vdad147 (PMC10681280; doi:10.1093/noajnl/vdad147)

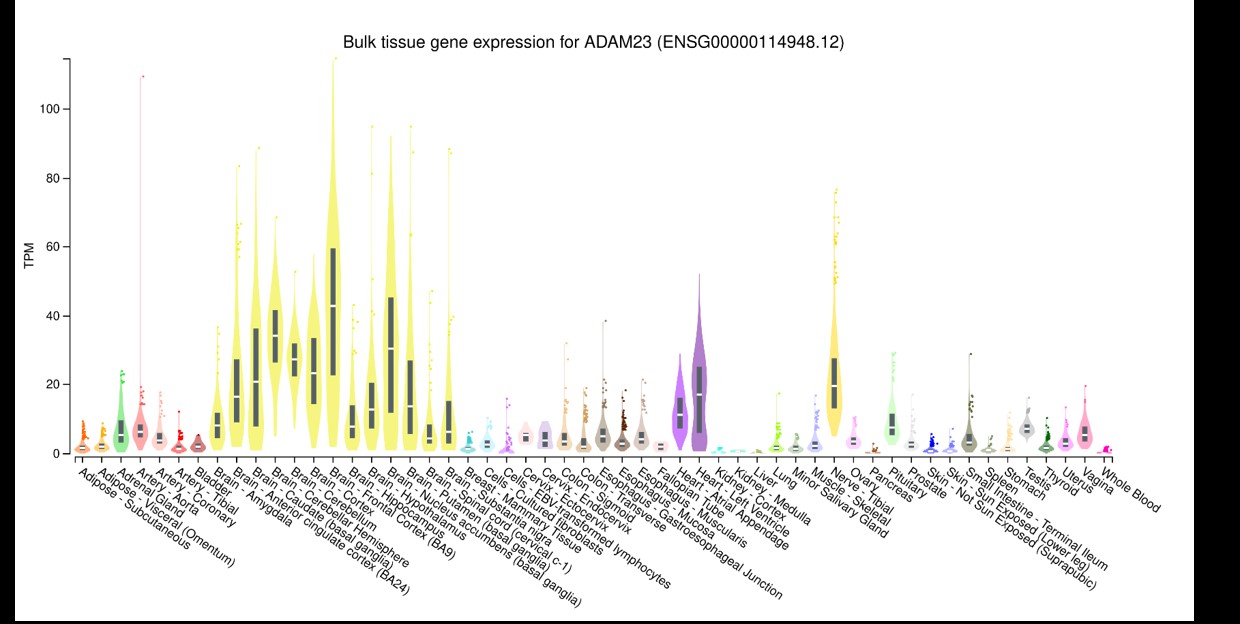

Supplement: vdad147_suppl_Supplementary_Figure_S1 [file vdad147_suppl_supplementary_figure_s1.jpeg]

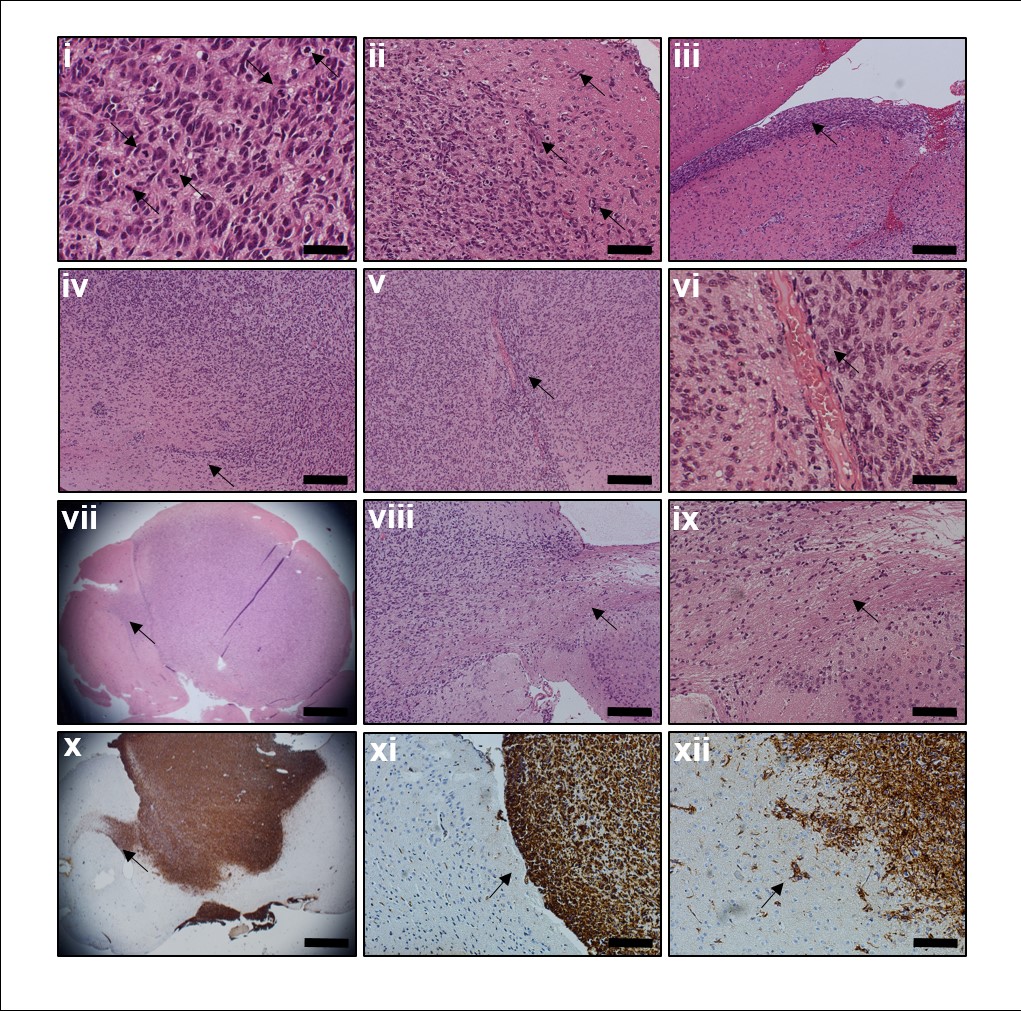

Supplement: vdad147_suppl_Supplementary_Figure_S2 [file vdad147_suppl_supplementary_figure_s2.jpeg]

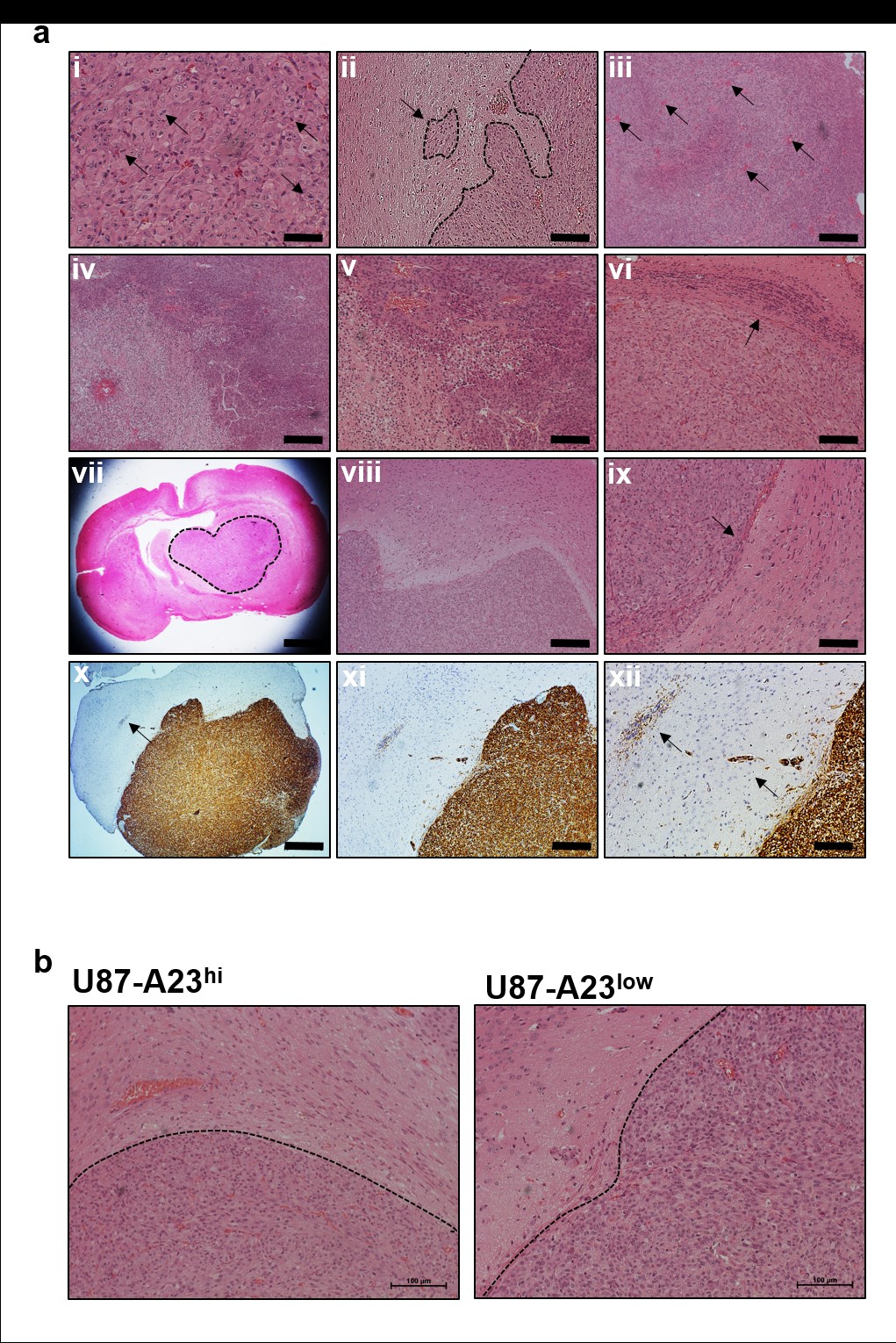

Supplement: vdad147_suppl_Supplementary_Figure_S3 [file vdad147_suppl_supplementary_figure_s3.jpeg]

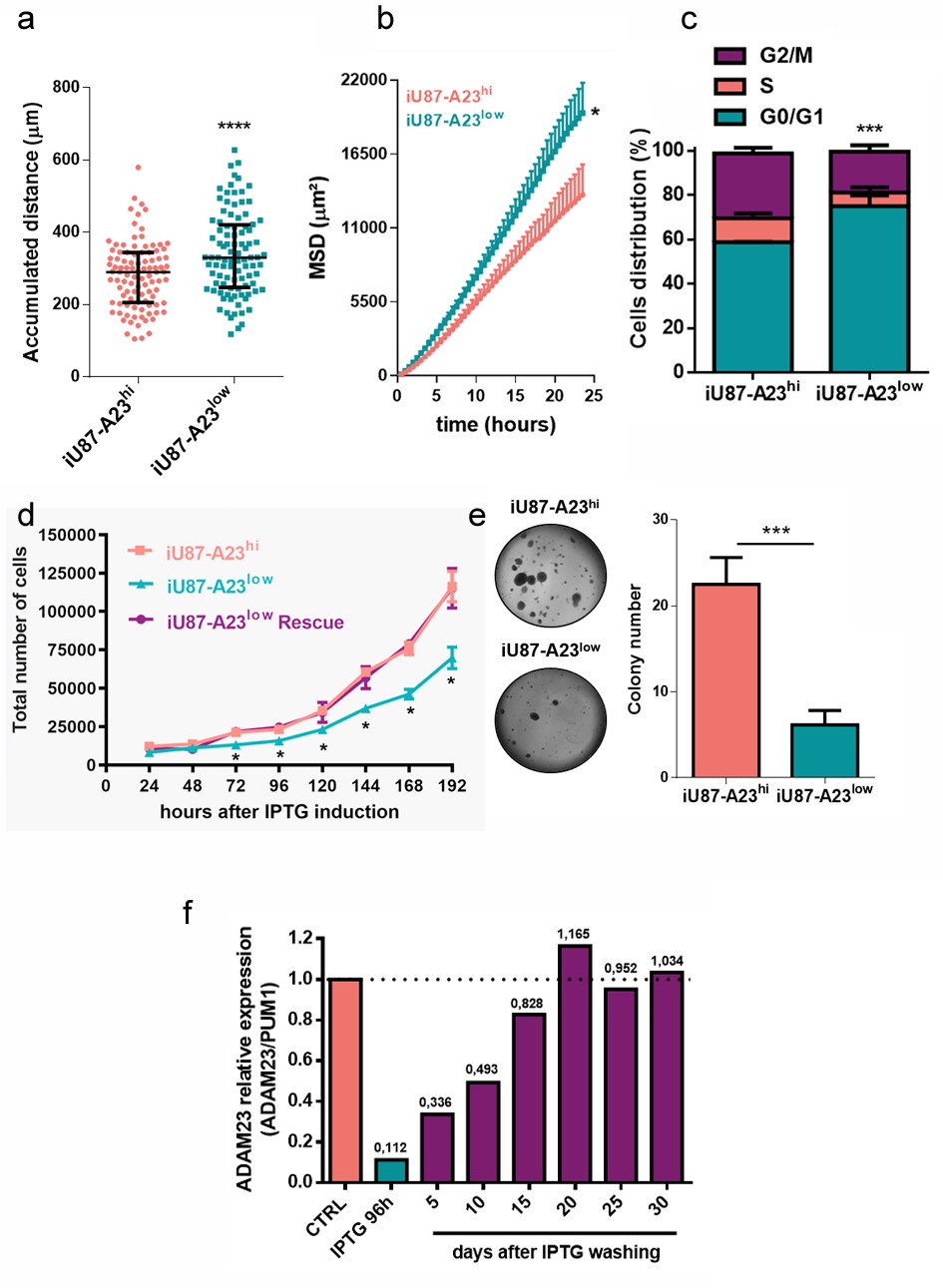

Supplement: vdad147_suppl_Supplementary_Figure_S4 [file vdad147_suppl_supplementary_figure_s4.jpeg]

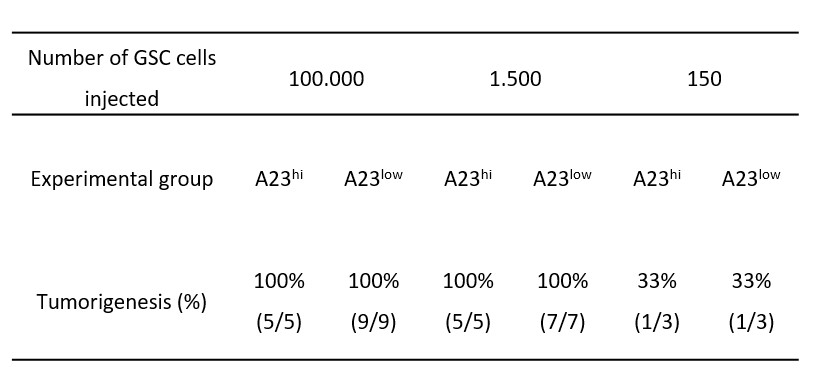

Supplement: vdad147_suppl_Supplementary_Figure_S5 [file vdad147_suppl_supplementary_figure_s5.jpeg]

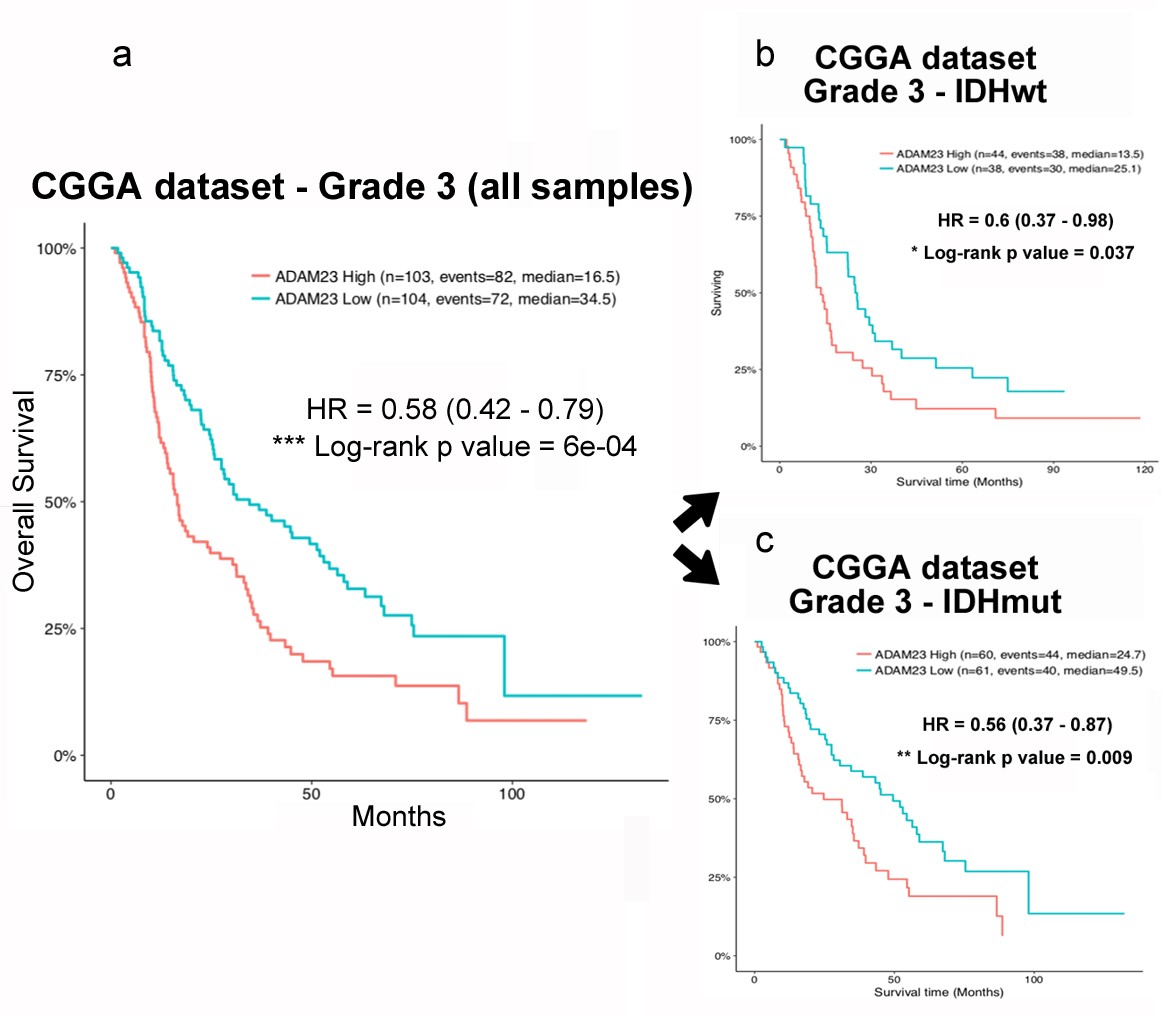

Supplement: vdad147_suppl_Supplementary_Figure_S6 [file vdad147_suppl_supplementary_figure_s6.jpeg]

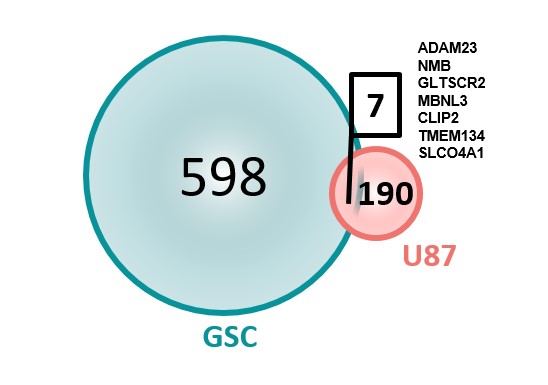

Supplement: vdad147_suppl_Supplementary_Figure_S7 [file vdad147_suppl_supplementary_figure_s7.jpeg]

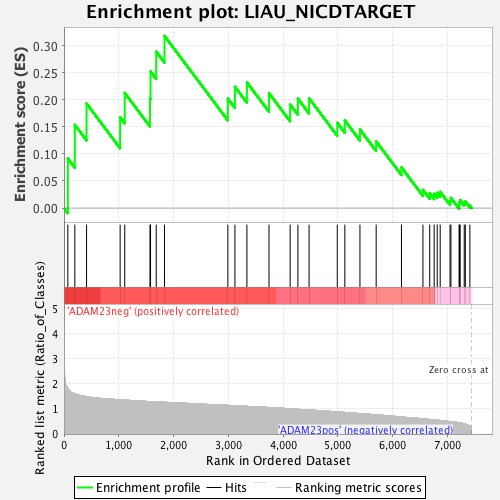

Supplement: vdad147_suppl_Supplementary_Figure_S8 [file vdad147_suppl_supplementary_figure_s8.jpeg]

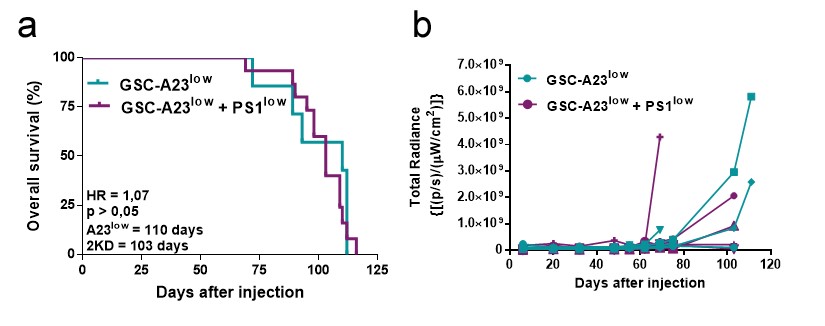

Supplement: vdad147_suppl_Supplementary_Figure_S9 [file vdad147_suppl_supplementary_figure_s9.jpeg]
