# Supplementary material for: A novel program of infiltrative control in astrocytomas: ADAM23 depletion promotes cell invasion by activating γ-secretase complex
Source: Neurooncol Adv. 2023 Nov 14;5(1):vdad147. doi: 10.1093/noajnl/vdad147 (PMC10681280; doi:10.1093/noajnl/vdad147)
Supplement: vdad147_suppl_Supplementary_Figures_Legends [file vdad147_suppl_supplementary_figures_legends.docx]

**SUPPLEMENTARY FIGURE LEGENDS**

**Fig S1. ADAM23 expression is enriched in normal brain tissue.** ADAM23 gene expression data and graph were obtained from the online GTEX platform.

**Fig S2. Histopathological features of GSC xenograft tumors in Balb/c Nude mice are from higher-grade astrocytomas.** i. Hematoxilin and Eosin (HE) staining in GSC tumors showing tumor high cellularity, anisotropy, and cell mitosis (black arrows); ii. GSC intense individual cell invasion in the normal brain parenchyma of the mice; iii. GSC subpial invasion; iv. GSC bulk cell migration; v-vi. Perivascular invasion; vii. Corpus callosum invasion; viii-ix. GSC invasion in white matter tracts; x-xii. GFAP staining showing Corpus callosum invasion, tumor retention in the brain hemisphere where the cells were inoculated, and single cell invasion in mice brain parenchyma.

**Fig S3. U87 xenograft tumors invasive growth pattern is ADAM23-independent. (A)** i. Hematoxilin and Eosin (HE) staining in U87 tumors showing tumor high cellularity, anisotropy, cell mitosis (black arrows), and an epithelial-like characteristics; ii. Representation of the extremely rare U87 collective invasion in the normal brain parenchyma of the mice; iii. High vascularization in U87 tumors (black arrows); iv-v. U87MG large areas containing intensive tumor necrosis. vi-ix. Tumor encapsulation and lack of interaction with mice brain parenchyma. x-xii. Vimentin staining showing perivascular invasion. **(B)** Absence of tumor invasion into mice brain parenchyma in A23^hi^ and A23^low^ U87 tumors.

**Fig S4. ADAM23 silencing augments iU87 mobility and diminishes cells proliferation. (A)** Accumulated distance travelled and **(B)** Mean Square Displacement (MSD) in iU87 A23^hi^ (n = 99) and A23^low^ (n = 96) cells for 24h in the hydrogel B-ECM in the absence (A23^hi^) and in the presence of 1mM IPTG (A23^low^). A and B data are represent as median, q25, and q75. Mann-Whitney test, * p < 0.05. **(C)** Cell cycle distribution analysis of iU87 cells after 96h of 1mM of IPTG. Data is represent as mean ± SD. Two-way ANOVA followed by Bonferroni’s post-test, *** p < 0.001. **(D)** Analysis of iU87 cell proliferation during seven days in the absence (A23^hi^) or in the presence of 1mM IPTG (A23^low^) and after 15 days of IPTG withdraw (A23^low^-Rescue). Data is represent as mean ± SD. Student’s t-test, * p < 0.05. **(E)** Anchorage-independent (soft-agar) clonogenic assay in the absence (A23^hi^) and in the presence of 1mM IPTG (A23^low^). Data is represent as mean ± SD (n = 4). Student’s t-test, * p < 0.05. **(F)** Kinetics of ADAM23 rescue by RT-qPCR analysis in iU87MG cells in the absence (CTRL, A23^hi^), in the presence of 1mM IPTG (IPTG 96h, A23^low^) and after up to 30 days of IPTG withdraw.

**Fig S5. ADAM23 silencing *in vivo* do not interfere with tumorigenesis.** **(A)** Tumorigenicity of GSC23 after the engraftment of 100,000, 1,500, and 150 cells in the right brain hemisphere of Balb/c Nude mice.

**Fig S6. ADAM23 expression is a prognostic marker in astrocytomas regardless IDH1 mutation status. (A)** Analyses of TCGA astrocytoma patients’ overall survival in grades 2-3 with IDH1 mutation and 1p/19q non-codelated (IDHmut non-codel) and **(B)** IDH wild-type (IDHwt). The overall survival was plotted in Kaplan-Meier curves and analyzed by Log-rank test.

**Fig S7. ADAM23 controls the expression of up-regulated AD-related genes. (A)** Venn-diagram showing the intersection between U87 and GSC DEGs.

**Fig S8. Transcriptional targets of NICD are not turned on when ADAM23 is downregulated.** GSEA of canonical Notch target genes in GSC with ADAM23^hi^ and ADAM23^low^ expression levels from Ref. 29.

**Fig S9. The co-silencing of PS1 gene with ADAM23 do not interfere with GSC23 tumorigenesis and mice survival.** **(A)** Mice survival after the engraftment of 1,500 GSC cells A23^low^ (n = 7) and A23^low^+PS1^low^ (n = 15) in the right striatum of Balb/c Nude mice. Kaplan-Meier analysis considering 20% of weight loss or neurological side effects as end-points. Log-rank test, p = 0.40. **(B)** Individual tumor growth kinetics (photons/ second) after the engraftment of 1,500 GS3 cells with A23^low^ and A23^low^+PS1^low^.
